# Incorporation of Copper Nanoparticles on Electrospun Polyurethane Membrane Fibers by a Spray Method

**DOI:** 10.3390/molecules28165981

**Published:** 2023-08-09

**Authors:** Tamer Al Kayal, Giulia Giuntoli, Aida Cavallo, Anissa Pisani, Paola Mazzetti, Rossella Fonnesu, Alfredo Rosellini, Mauro Pistello, Mario D’Acunto, Giorgio Soldani, Paola Losi

**Affiliations:** 1 Institute of Clinical Physiology, National Research Council, 54100 Massa, Italy; giuntoli.g@ifc.cnr.it (G.G.); aida.cavallo@ifc.cnr.it (A.C.); apisani@ifc.cnr.it (A.P.); giorgio.soldani@ifc.cnr.it (G.S.); losi@ifc.cnr.it (P.L.); 2 Virology Unit, Pisa University Hospital, 56124 Pisa, Italy; p.mazzetti@ao-pisa.toscana.it (P.M.); fonnesurossella@gmail.com (R.F.); al.rosellini@ao-pisa.toscana.it (A.R.); mauro.pistello@unipi.it (M.P.); 3 Retrovirus Center, Department of Translational Research and New Technologies in Medicine and Surgery, University of Pisa, 56124 Pisa, Italy; 4 Institute of Biophysics, National Research Council, 56124 Pisa, Italy; mario.dacunto@ibf.cnr.it

**Keywords:** electrospinning/spray, copper nanoparticles, polyurethane fibers, antibacterial, antiviral

## Abstract

Electrospinning is an easy and versatile technique to obtain nanofibrous membranes with nanosized fibers, high porosity, and pore interconnectivity. Metal nanoparticles (e.g., Ag, Cu, ZnO) exhibit excellent biocide properties due to their size, shape, release of metal ions, or reactive oxygen species production, and thus are often used as antimicrobial agents. In this study, a combined electrospinning/spray technique was employed to fabricate electrospun polyurethane membranes loaded with copper nanoparticles at different surface densities (10, 20, 25, or 30 μg/cm^2^). This method allows particle deposition onto the surface of the membranes without the use of chemical agents. SEM images showed that polyurethane fibers own homogeneous thickness (around 650 nm), and that spray-deposited copper nanoparticles are evenly distributed. STEM-EDX demonstrated that copper nanoparticles are deposited onto the surface of the fibers and are not covered by polyurethane. Moreover, a uniaxial rupture test showed that particles are firmly anchored to the electrospun fibers. Antibacterial tests against model microorganisms *Escherichia coli* indicated that the prepared electrospun membranes possess good bactericidal effect. Finally, the antiviral activity against SARS-CoV-2 was about 90% after 1 h of direct contact. The obtained results suggested that the electrospun membranes possess antimicrobial activities and can be used in medical and industrial applications.

## 1. Introduction

Electrospinning is a simple and versatile method widely used for the production of membranes loaded with antibacterial and antiviral nanoparticles (NPs) for medical and environmental applications [1,2,3].

Electrospun membranes (EM) are characterized by high porosity and a high surface area to volume ratio; these properties allow for better exposure of the bioactive NPs.

For NPs’ incorporation in EM different approaches are commonly used: (a) direct incorporation, such as blend electrospinning, where NPs are added to polymer solution before the electrospinning process [4,5,6], or in situ synthesis of NPs, where the precursor is added to the polymer solution so that NPs’ nucleation and growth occurs during electrospinning concurrently with nanofiber formation [7,8], or (b) post-modification techniques, such as surface modifications (e.g., plasma treatments, chemical functionalization, surface grafting), physical deposition (e.g., sputtering, evaporation, or thermal deposition), layer-by-layer assembly, immersion, or dip coating, where the NPs are added onto the EM by chemical or physical treatments [9,10].

Various metal and metal oxide particles have been used as antibacterial or antiviral agents for the functionalization of EM, such as silver, iron oxide, titanium dioxide, lanthanum oxide, zinc oxide, and copper. NPs’ mechanisms against numerous pathogens include: (i) contact-killing abilities, due to their small size and large surface area which facilitate interactions with bacterial cell walls, leading to structural damages and leakage of cellular components; (ii) reactive oxygen species (ROS) generation, that can cause oxidative stress, disrupting cellular processes, and damaging bacterial DNA, proteins, and cell membranes; (iii) ion release, that interferes with cellular processes and replication cycles, thus inhibiting bacterial growth [11,12,13].

Although silver has been the most widely used antimicrobial agent in both medical and industrial applications due to its broad-spectrum antimicrobial properties [14], there are emerging concerns regarding the extensive use of silver NPs, and some researchers suggest that this may lead to the appearance of Ag-resistant bacteria [15]. With the emergence of the COVID-19 pandemic, several studies have instead focused on the use of copper as a bioactive agent due to its high antimicrobial activity against bacteria and viruses, and its low cost compared to silver [16,17,18,19].

A study carried out in the initial period of the pandemic has shown that SARS-CoV-2 virus is inactivated after 4 h of contact with a copper surface, and SARS-CoV-1 virus is inactivated after 8 h [20]. It has also been shown that the size of copper particles has an important role in the antimicrobial capacity of copper: nano-sized Cu or CuO particles showed a higher degree of antimicrobial activity compared with micrometer-sized particles [21].

For bioactive EM preparation with copper nanoparticles (CuNPs), different polymers have been studied according to their final application. For example, for wound-dressing applications, a polycaprolactone/chitosan membrane was prepared by blend electrospinning with copper particles; the obtained EM showed a bactericidal capacity [22]. Moreover, a polyurethane (PU) membrane was prepared for air filtration system with micro- and nano-sized copper particles dispersed in the electrospinning solution. In this case, the obtained membrane also showed an antimicrobial capacity [5,18]. PUs are a versatile class of polymers widely used in the medical field due to their excellent mechanical properties, durability, hemocompatibility and biocompatibility [23].

In this study, we report an innovative and easy spray method for the functionalization of PU electrospun membranes (PU-EM) with CuNPs. Furthermore, the biocompatibility, bactericidal and virucidal capacity of the functionalized membranes has been demonstrated. Due to the obtained results, the PU-EM loaded with CuNPs produced with our method could be straightforwardly applied as membranes with antibacterial/antiviral activities for medical and industrial applications.

## 2. Results

### 2.1. Morphological Characterization

**Figure 1:**
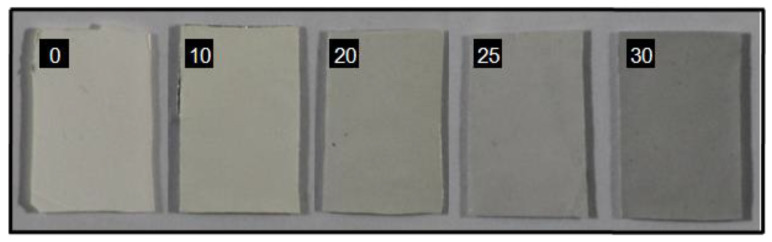
Macroscopic images of pristine polyurethane electrospun membranes (PU-EM, 0 μg/cm^2^), and PU-EM loaded with CuNPs at different surface densities, from 10 to 30 μg/cm^2^.

Macroscopic observation of the PU-EM samples loaded with CuNPs (PU-EM-CuNPs) (Figure 1) shows a homogeneous distribution of the gray color on each membrane. Furthermore, an increase in color intensity is observed with the increase in the surface densities of CuNPs.

**Figure 2:**
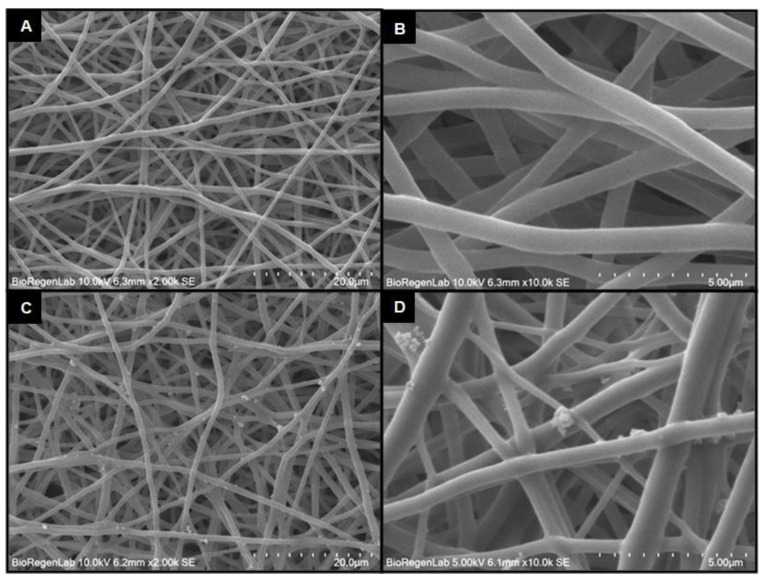
SEM images of polyurethane electrospun membranes (PU-EM) at different magnifications. (**A**,**B**) Pristine PU-EM, and (**C**,**D**) PU-EM functionalized with 30 μg/cm^2^ of CuNPs.

The scanning electron microscopy (SEM) images of the samples show that the control fibers are smooth and continuous, with diameters of 644 ± 134 nm (Figure 2A,B). The fabrication of PU-EM by electrospinning is a reproducible process, as confirmed by the analysis of fibers diameters. Furthermore, SEM images of PU-EM-CuNPs show a homogeneous distribution of the particles on the PU-EM with the formation of some small aggregates (Figure 2C,D). To assess the impact of ethanol (EtOH) on PU fibers, the analysis of the thickness of the fibers was conducted following EtOH spray (without CuNPs). The results did not show significant variations between the control PU-EM and the samples treated with EtOH, which presented an average diameter of 656 ± 146 nm, similar to untreated PU-EM fibers.

### 2.2. Qualitative Assessment of CuNPs Adhesion and Stability

**Figure 3:**
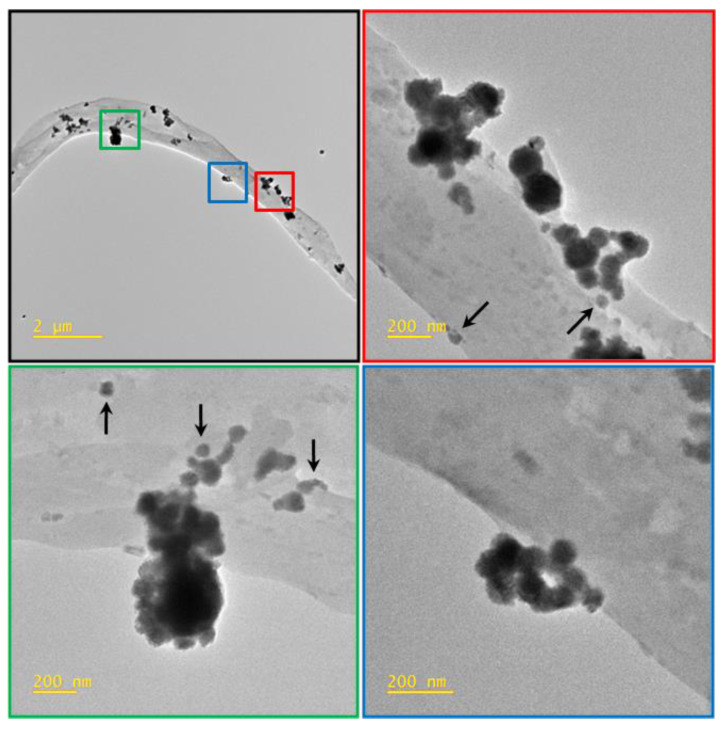
Bright Field TEM image of polyurethane electrospun membranes loaded with CuNPs; arrows indicate single nanoparticles.

The TEM images (Figure 3) of PU-EM-CuNPs samples show the presence of CuNPs embedded on the surface of the PU fibers. Furthermore, a distribution of the particles in clusters is observed as they tend to aggregate during the spray deposition process. There are also clearly visible individual particles immobilized on the surface of the fiber, whereas no particles fully embedded within the fibers are observed.

**Figure 4:**
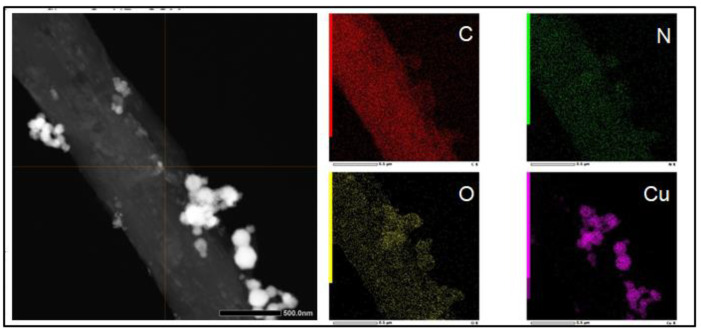
STEM-EDX elemental maps for Carbon, Nitrogen, Oxygen and Copper of polyurethane electrospun membranes (PU-EM) loaded with CuNPs.

The STEM-EDX analysis for Carbon, Nitrogen, Oxygen, and Copper elements showed that the CuNPs are not covered by the PU matrix (Figure 4, C), indicating that the particles are exposed to the external environment. Moreover, the presence of an oxidized form of copper is revealed (Figure 4, O).

**Figure 5:**
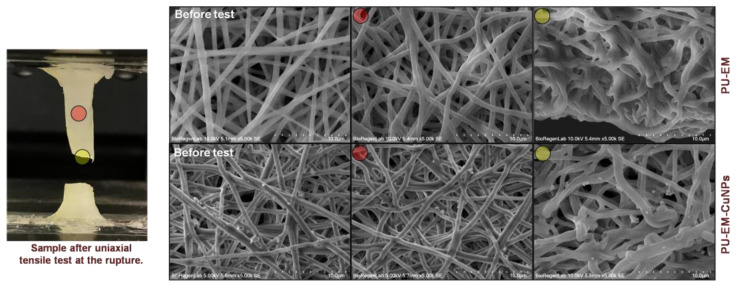
(**Left**): macroscopic image of a sample after the uniaxial tensile test. (**Right**): SEM images at different magnifications of pristine polyurethane electrospun membranes (PU-EM) (**top** row), and PU-EM loaded with 30 μg/cm^2^ of CuNPs (PU-EM-CuNPs) (**bottom** row).

To evaluate the adhesion of CuNPs to the surface of PU fibers, SEM analysis of a PU-EM-CuNPs membrane subjected to uniaxial tensile test was performed (Figure 5). The examination was carried out after fracture occurred. SEM images show that, after mechanical stretching, the particles are firmly anchored to the deformed fibers throughout the sample. Even at the point of fracture, after experiencing significant elongation (around 230% strain), the particles remain bound to the polymeric surface. Qualitatively, this demonstrates that sprayed CuNPs are well-adhered to the polymeric fibers.

**Figure 6:**
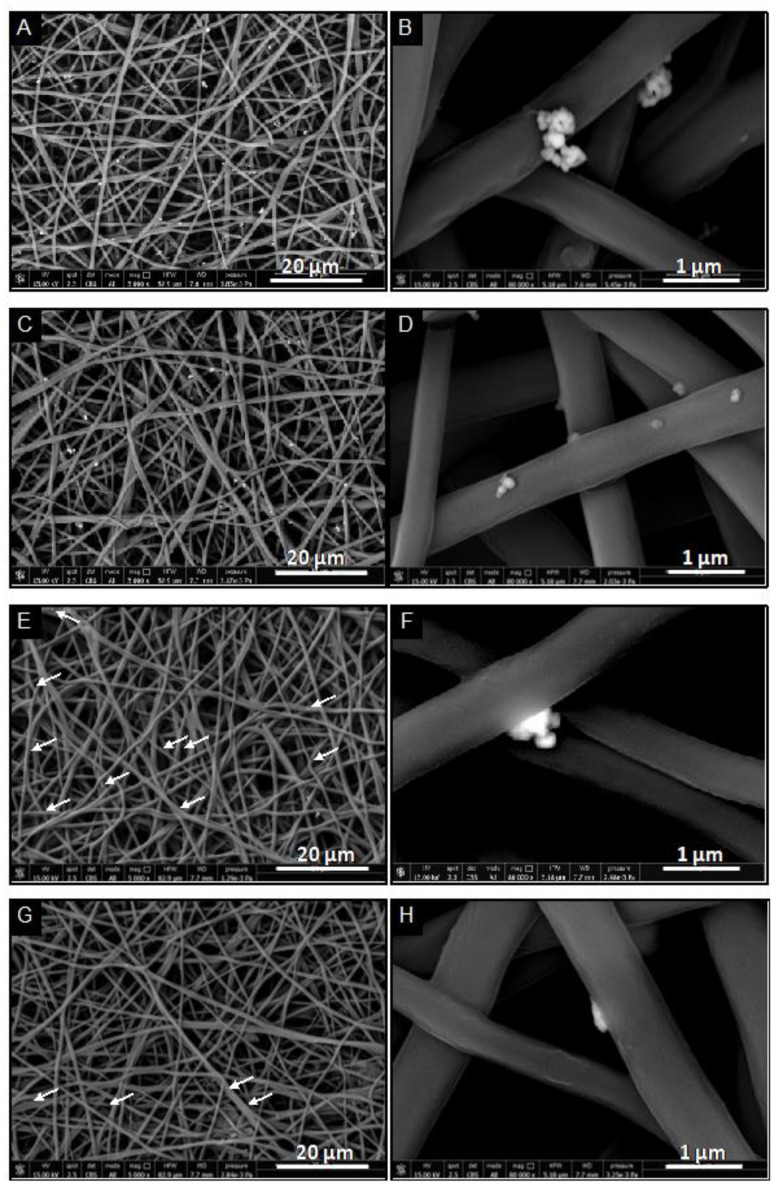
Representative SEM images at different magnifications of polyurethane electrospun membranes (PU-EM-CuNPs) loaded with 30 μg/cm^2^ of CuNPs at (**A**,**B**) t = 0, or after 24 h incubation at 37 °C in (**C**,**D**) dH_2_O, (**E**,**F**) PBS, or (**G**,**H**) RPMI+ cell culture medium. White arrows in (**E**,**G**) indicate CuNPs.

Moreover, the evaluation of the stability and dissolution of CuNPs was performed by incubating PU-EM-CuNPs samples in different media at 37 °C under agitation, over a period of 24 h. SEM observations (Figure 6) revealed that CuNPs are stable in dH_2_O and remained attached to the PU fibers (Figure 6C,D), being comparable to the control (Figure 6A,B), whereas they are almost completely released in a PBS or in RPMI+ cell culture medium, although few sparse nanoparticles could be observed (white arrows in Figure 6E,G).

### 2.3. Biocompatibility Evaluation by the Indirect Contact Method

**Figure 7:**
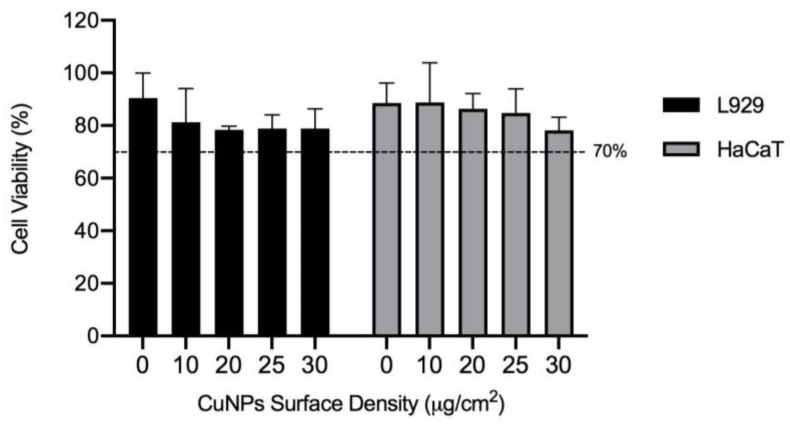
In vitro biocompatibility by indirect contact method of polyurethane electrospun membranes (PU-EM) (0 μg/cm^2^), and PU-EM samples loaded with 10, 20, 25, or 30 μg/cm^2^ of CuNPs evaluated by MTT assay with L929 or HaCaT cells over 24h of culture. Histograms represent the mean ± standard deviation (n > 3).

The possible cytotoxic effect of samples was evaluated by an indirect cytotoxicity test against either L929 or HaCaT cells, through the MTT assay. Figure 7 shows that the viability of both cell types is above 70% for all CuNPs concentrations tested, displaying no indication of cytotoxicity due to the presence of CuNPs. PU-EM control samples are highly biocompatible, with 90 ± 9% and 86 ± 7% viability for L929 and HaCaT cells, respectively, proving that no residual organic solvents remain after the electrospinning process.

The addition of CuNPs onto the surface of PU-EM by the spray deposition process induced a slight but not significant decrease in L929 cell viability as a function of CuNPs content, reaching a minimum viability of 78%. The same trend was observed for HaCaT cells, for which the minimum viability measured was 77%.

### 2.4. Antibacterial Activity against E. coli

**Figure 8:**
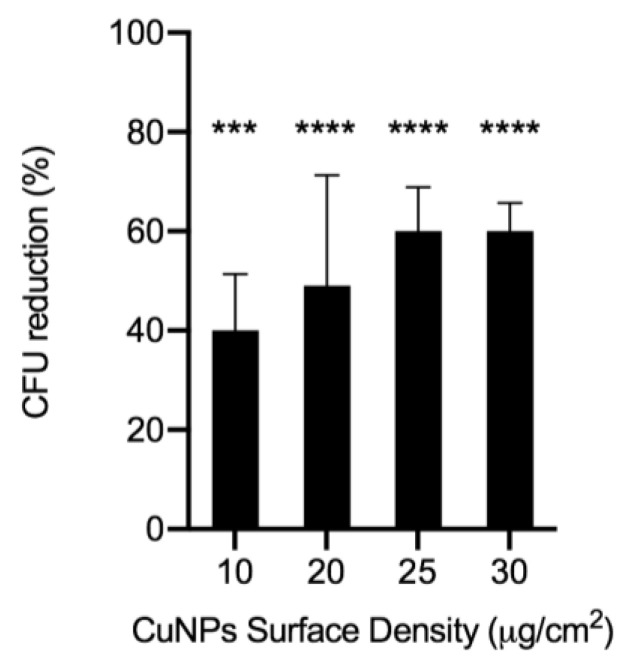
Antibacterial activity against *E. coli* of polyurethane electrospun membrane samples loaded with 10, 20, 25, or 30 μg/cm^2^ of CuNPs. Histograms represent the mean ± standard deviation (n > 3). *** *p* <0.0005 and **** *p* <0.0001 when compared to the control (membrane without CuNPs).

The antibacterial activity against *E. coli* of PU-EM-CuNPs samples was evaluated by the direct contact method. Figure 8 shows that the fibers loaded with 25 and 30 μg/cm^2^ CuNPs exerted a relevant antibacterial activity against *E. coli* giving more than 50% of CFU reduction.

### 2.5. Antiviral Activity against SARS-CoV-2

**Figure 9:**
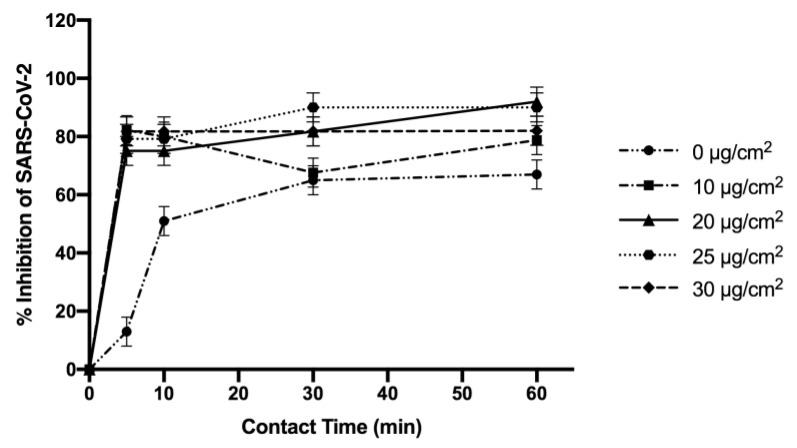
Inhibition of SARS-CoV-2 by polyurethane electrospun membranes (PU-EM) (0 μg/cm^2^), and PU-EM samples loaded with CuNP sat different surface densities.

The results of Coronavirus SARS-CoV-2 inhibition at different time points of contact (5, 10, 30, and 60 min) are illustrated in Figure 9. All samples showed a virus inhibition activity of around 80% after 5 min of contact and a maximum of SARS-CoV-2 inhibition of 90% was obtained with PU-EM-CuNPs at 20 and 25 µg/cm^2^ after 60 min of contact.

## 3. Discussion

This study aimed at the production of polyurethane electrospun membranes loaded with CuNPs with antibacterial and virucidal properties by a new method based on a spray process.

Copper has been used in its different forms (powder, metal surface, micro or nano- particles) as an antibacterial and virucidal agent [24].

A conventional method to obtain EM functionalized with NPs is by direct incorporation of NPs through blend electrospinning or by in situ synthesis of NPs using precursors. However, these methods have some major drawbacks, for instance: agglomeration of NPs, which can lead to non-uniform distribution within the nanofibers; loss of functionality, due to the intense electric field that particles experience during electrospinning; reduced loading capacity of NPs, to ensure adequate solution processability. Moreover, with blend electrospinning only a few particles would be exposed on the surface and most of them would remain trapped inside the fibers, losing their effectiveness as antibacterial agents [6]. Recently, Ungur and colleagues developed electrospun PU matrices with Cu nano- and micro-particles via blend electrospinning. SEM analysis showed that the loaded Cu nanoparticles formed large aggregates in the electrospun fibers, due to the low dispersion of the particles in solution which tended to aggregate, leading to an increase in the viscosity of the electrospun solution [5]. Besides aggregation, when the particles employed during blend electrospinning have dimensions smaller than the diameter of the nanofibers, the majority of the particles will tend to be completely incorporated within the polymeric fiber. As previously observed, the few particles that could be found at the surface will be covered with a polymeric layer [6,25]. Other studies report that the addition of metal particles in the polymeric solution leads to fibers with dimensions different from those of the original polymer due to the effect of the particles increasing both the solution viscosity, but mainly its conductivity [5,25]. Further, the presence of NPs can negatively impact on EM thermal stability and mechanical properties [8].

Among post-modification techniques, spray deposition offers several advantages such as simplicity, versatility, and the ability to coat large areas or complex-shaped substrates [26]. Moreover, it allows for a relatively uniform and controlled deposition of NPs onto the nanofiber surface [27]. During spray deposition, the air pressure-atomized spray droplets carrying the NPs undergo drying or solvent evaporation, leading to the NPs’ attachment or embedding within the fibers upon contact [27].

Herein, we successfully obtained PU-EM-CuNPs with increasing surface densities of CuNPs, as can be straightforwardly seen by visual inspection. The different loadings of CuNPs were obtained by adjusting the initial CuNPs concentration (150, 300, 400, 500 µg/mL in EtOH) while maintaining the spraying duration as a constant, so that the same volume of EtOH was used for each PU-EM-CuNPs membrane. Indeed, it was also assessed that EtOH does not alter the original morphology and diameters of PU-EM fibers. SEM and TEM analyses of PU-EM-CuNPs samples showed that all spray-deposited CuNPs were found at the surface of the fibers, and no CuNPs were embedded within the polymeric fibers; in addition, CuNPs exhibited sustained contact with PU fibers even after mechanical stretching and sample fracture, demonstrating robust adhesion despite potential interfacial shear caused by strain mismatch. Similarly, Aminu et al. employed the same method to qualitatively assess the adhesion of copper nanocubes on polyacrylonitrile fibers after mechanical stretching [9]. STEM-EDX confirmed that the CuNPs were not coated with a polymeric layer, so that they were exposed to the environment, maximizing their biological activity. Elemental mapping further revealed an oxidized layer on CuNPs, which could derive from metallic Cu instability and inherent reactivity with oxygen in the atmosphere, resulting in the formation of an oxidized layer [28]. This phenomenon of partial oxidation of commercial CuNPs after exposure to air has been previously documented [29]. Although some aggregates were observed, they were of small dimensions, as compared to those observed by Ungur [5,18]. This phenomenon could derive from a partial evaporation of the solvent during the spray deposition process, and from the natural tendency of CuNPs to aggregate. Although CuNPs dispersion was previously sonicated before use, CuNPs inside atomized droplets may have started to agglomerate [27].

To assess the suitability of PU-EM-CuNPs as antimicrobial coatings in various fields, including medical and healthcare, food packaging, water filtration, and air purification systems, indirect cytotoxicity evaluations were carried out. An L929 mouse fibroblast cell line is conventionally used for biocompatibility evaluations [30], while HaCaT is a human epidermal keratinocytes line and was chosen as a representative cell model due to the potential use of PU-EM-CuNPs as wound-dressing materials, nonwoven materials for surgical masks and personal protective equipment production, or other applications that imply a direct contact of the membrane with the skin. As for other metal or metal-oxide particles, such as silver NPs, the cytotoxic effect of CuNPs depends on their size, shape, and concentration [21,31,32,33]. For instance, Na and Kennedy tested the cytocompatibility of CuNPs towards three human cell lines and reported that particles of 40–60 nm are cytotoxic due to a high cellular intake, while bigger or smaller particles are less up-taken but cause higher oxidative stress [31]. Similarly, Semisch et al. proved that CuO micro-particles exerted no cytotoxicity in a range up to 50 µg/mL, while CuO NPs have a pronounced cytotoxic effect [32]. In our work, the spray deposition process allowed us to obtain well-dispersed CuNPs on the surface of PU-EM, although some degree of aggregation of CuNPs could be observed. We could hypothesize that the strong attachment of CuNPs to the underlying network and the amount of released Cu ions contributed to the high biocompatibility of the prepared samples, even for samples with the highest content of CuNPs (30 μg/cm^2^) [34]. Midander et al. evaluated the toxicity against lung cells of nano- and micro-sized metallic Cu particles and reported a strong cytotoxic effect on cells directly exposed to CuNPs, whereas the cytotoxicity induced by the released Cu fraction was significantly lower [21]. Our results seem to be in agreement with these observations. In fact, the evaluation of the stability of PU-EM-CuNPs after 24 h incubation proved that CuNPs are stable in dH_2_O, whereas the majority of CuNPs are dissolved/released in PBS or RPMI+, as previously reported [21,35]. Therefore, in our case, the amount of released copper, in the form of either ions or nanoparticles, is not cytotoxic and only causes a slight reduction in cell viability. We did not observe any strong correlation between the cell viability and content of the CuNPs, in the range studied.

The antibacterial mechanisms of CuNPs have not yet been fully elucidated; however, they include contact-killing abilities, cell membrane damage through electrostatic interactions or ROS, unbalance of metal/metal ions homeostasis, and interference with replication cycles [11,19]. Several studies have demonstrated good antibacterial activity of electrospun PU membranes loaded with copper particles by blend electrospinning [5,18,36]. These studies used concentrations between 5 and 12% of copper nano- or micro-particles, relative to the polymer content. In our research, the theoretical concentration of CuNPs on the membrane varies from 0.2% to 0.6% for PU-EM-CuNPs loaded with 10 µg/cm^2^ or 30 µg/cm^2^ of CuNPs, respectively. Ungur and colleagues report that the antibacterial activity for the PU membrane containing 12% of CuNPs after 2 h of contact was between 50% and 65% against *E. coli* [5]. The antibacterial activity results obtained in this study after 2 h of direct contact with *E. coli* showed an efficacy of ~45% for 20 µg/cm^2^ samples, and ~60% for 25 and 30 µg/cm^2^ samples (corresponding to 0.5 and 0.6% *w*/*w* of CuNPs). Therefore, these results demonstrate that the proposed method allows a drastic decrease in the amount of CuNPs needed to achieve an efficient antibacterial effect compared to the blend electrospinning method, due to a superficial particle distribution and the absence of large aggregates. In their study on the antibacterial efficacy of different commercial silver wound dressings, Gallant-Behm et al. demonstrated that there is no correlation between the zone of inhibition (ZOI) and log reduction data; for instance, the non-releasing silver dressing Actisorb Silver 220 (composed of TNT impregnated with 33 µg/cm^2^ of Ag) did not show any ZOI for the microorganisms tested, but resulted effective in killing gram-negative bacteria by the log reduction test, highlighting the limitations of disk-diffusion assays [37]. Contrarily, other studies on EM functionalized with CuNPs report clear ZOI due to the controlled release of CuNPs from the nanofibers [22]. Our results suggest that there is a simultaneous activation of multiple antibacterial mechanisms, such as ion release and contact killing, each one contributing to the toxicity towards pathogens [19]. For the proposed applications, PU-EM-CuNPs membranes provide adequate antibacterial activity employing very low concentrations of CuNPs.

With the emergence of COVID-19, the need for antiviral substrates and coatings had arisen. Since nanomaterials have strong antiviral properties [38], their use was largely evaluated to arrest the spread of SARS-CoV-2.

In a previous study, a spray method was used to obtain a coating of surgical masks with a blend of 0.5 *w*/*v* polyurethane and 1% CuNPs *w*/*v* (200% in relation to the polymer) to obtain antiviral activity against SARS-CoV-2 [39]. The results showed a virucidal activity of 94% after 10 min of contact with the virus and 100% after 60 min. Our samples prepared with NPs densities of 20 and 25 µg/cm^2^ by the electrospinning-spray method showed a reduction in the virucidal activity of 90% after 60 min of contact with the virus. This demonstrates that the choice of electrospun membranes and the CuNPs spray loading have allowed in this case a reduction in the amount of particles used. In addition, it is important to note that PU materials do not possess inherent antiviral properties [40]. Therefore, the observed reduction in virus titer for PU-EM control samples is likely to be attributable to the nanofibrous and porous structure of the membrane. This structure enables the capture and retention of small pathogens such as viruses, thereby enhancing the antimicrobial properties, particularly when combined with CuNPs.

These preliminary data highlight the potential of this loading method for the preparation of PU-EM-CuNPs membranes against pathogens in both environmental and medical fields.

## 4. Materials and Methods

### 4.1. Materials

Thermoplastic aromatic poly(ether)urethane Estane^®^ 5714F1 was purchased from Lubrizol Advanced Materials (Wickliffe, OH, USA). Metal CuNPs powder (average particle size 60–80 nm, purity 99.9%) was purchased from IoLiTec Nanomaterials (Heilbronn, Germany). Tetrahydrofuran (THF), dimethylformamide (DMF), ethanol, methanol, and acetone were obtained from CarloErba Reagents s.r.l. (Val de Reuil, France)

Mouse fibroblasts cell line L929 (ICLC ATL95001) was obtained from Biobanking and Cell Factory Hospital San Martino (Genova, Italy). Human keratinocytes HaCaT (BS CL 168) were obtained from Biobanking of Veterinary Resources, Istituto Zooprofilattico Sperimentale della Lombardia e dell’Emilia Romagna (Brescia, Italy). *Escherichia coli* was obtained from Thermofisher (Milan, Italy). Cell and bacteria culture reagents (RPMI 1640, L-Glutamine, fetal bovine serum (FBS), streptomycin and penicillin, phosphate buffer saline, 3-(4,5 dimethylthiazol-2-yl)-2,5-diphenyl tetrazolium bromide (MTT), dimethyl sulfoxide (DMSO) reagent, Muller–Hinton broth) were purchased from Merck KGaA (Darmstadt, Germany).

### 4.2. Fabrication of CuNPs-Loaded Electrospun Membranes

**Figure 10:**
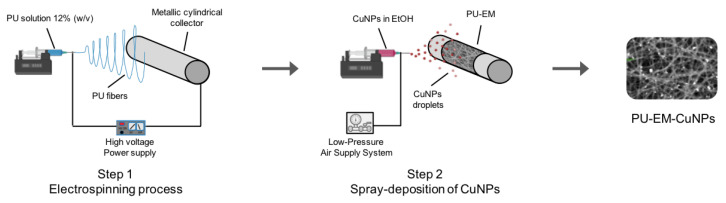
Schematic of polyurethane (PU) electrospun membranes (PU-EM) functionalized by CuNPs via a spray-deposition process.

The fabrication process is divided into two phases, as schematized in Figure 10.

- Step 1: Procedure for PU-EM

PU pellets were first subjected to purification by a soxhlet apparatus in a methanol–acetone mixture (1:1 *v*/*v*). Then, PU pellets were dissolved in a mixture of THF, DMF, and dH_2_O (58:40:2 *v*/*v*) to a final concentration of 12% *w*/*v*. The PU solution was then loaded into a 10 mL glass syringe with a 19G metallic needle and electrospun (Linari Engineering S.r.l., Pisa, Italy) onto a metallic cylindrical collector (50 mm diameter) covered with aluminum foil at room temperature. For each PU-EM membrane, 5 mL of solution were electrospun. The following electrospinning parameters were used: applied voltage 22 kV, flow rate 1 mL/h, working distance 21 cm, collector rotation 125 rpm. For each PU-EM membrane, 5 mL of solution were electrospun (5 h collection time).

- Step 2: Spray deposition of CuNPs on PU-EM

The spray-machine apparatus used in this work is custom equipment that consists of separate ejectors (spray guns) mounted onto a sliding carriage, a low-pressure air supply system, a remote-control unit, and rotating collectors.

For the spray-deposition process, commercial CuNPs were first dispersed in 10 mL ethanol at different concentrations (150, 300, 400, 500 µg/mL); then, CuNPs dispersions were ultrasonicated (frequency 59 kHz, power 100%) for at least 40 min in a sonication bath. After this, the dispersions were loaded into a plastic syringe and sprayed onto the PU-EM by setting the following parameters: flow rate 40 mL/h, gun pressure 12 psi, working distance 54 mm, collector rotation 88 rpm, carriage speed 23.3 mm/s and carriage translation 100 mm along the x-axis. During the spray process, aspiration inside the spray chamber was on. After CuNPs spray deposition, the aluminum foil was detached from the collector and left under a hood overnight.

The CuNPs-loaded PU-EM are named PU-EM-CuNPs. The final CuNPs surface densities of CuNPs were 10, 20, 25, and 30 μg/cm^2^. To remove unattached CuNPs, the samples were washed thrice in agitation (40 rpm) in dH_2_O for a total of 90 min. PU-EM samples were used as controls. At least three samples for each membrane type were prepared.

### 4.3. Morphological Characterization

The morphology of PU-EM and PU-EM-CuNPs samples was observed using a scanning electron microscope (SEM, FlexSEM 1000, Hitachi, Tokyo, Japan), at different magnifications, with an accelerating voltage of 5 or 10 kV. All samples were previously sputter-coated with gold. SEM images were analyzed with ImageJ 1.51K software (National Institutes of Health, Bethesda, MD, USA), to estimate the average fiber diameters from at least three distinct membranes (three areas per membrane, 50 measures per image).

### 4.4. Qualitative Assessment of CuNPs Adhesion and Stability

#### 4.4.1. Transmission Electron Microscopy (TEM)

TEM images were collected using a ZEISS Libra 120 TEM (Carl Zeiss NTS, Oberkochen, Germany), operating at an accelerating voltage of 120 kV and equipped with an in-column omega filter. Samples were put on 300-mesh carbon-coated copper grids and dried before observation. Energy-dispersive X-ray spectroscopy (EDX) was performed with a JEOL EM-F2000 Multi-purpose, working at 200 kV and equipped with a Schottky-FEG source and high-counting SDD EDX detector. EDX maps were performed in scanning mode, with a probe size of 1 and a counting time of 2 min.

#### 4.4.2. Uniaxial Tensile Test

Uniaxial tensile test was performed to qualitatively evaluate the adhesion of CuNPs to the polymeric PU-EM matrix. Tests were performed on dog-bone shaped samples (5 mm width, 15 mm gauge length) with a Zwick-Roell Z1.0 testing machine (Zwick GmbH & Co., Ulm, Germany) equipped with a 100 N load cell, at a crosshead speed of 10 mm/min. Samples were mounted using screw grips that prevented slippage during elongation, and tested until rupture. After the test, the samples were washed thrice in agitation (40 rpm) in dH_2_O for a total of 90 min and were analyzed by SEM.

#### 4.4.3. CuNPs Stability in Different Media

The stability and dissolution of CuNPs deposited on PU-EM was evaluated in different media, namely dH_2_O, PBS, or RPMI+ cell culture medium (RPMI 1640 supplemented with 10% FBS, 1% of 2 mM L-Glutamine, 100 µg/mL streptomycin and 100 U/mL penicillin). Samples were incubated in the different media (1 mL/3 cm^2^) at 37 °C under agitation for 24 h. After the test, the samples were washed thrice in agitation (40 rpm) in dH_2_O for a total of 90 min and were analyzed by SEM.

### 4.5. Biocompatibility Evaluation by the Indirect Contact Method

In vitro cytotoxicity evaluations were carried out on L929 and HaCaT cells. Cells were routinely cultured in RPMI+ at 37 °C and 5% CO_2_; medium was changed on average every 3 days. Before the test, samples (15 × 10 mm) were sterilized under UV light for 1 h per side. The conditioned media were obtained by incubating samples in RPMI+ (1 mL/3 cm^2^) at 37 °C under agitation for 24 h. To determine cell viability, the MTT colorimetric assay was employed. Briefly, L929 mouse fibroblasts or HaCaT human keratinocytes were collected by trypsinization and seeded in 96-well plates at 1 × 10^4^ and 2 × 10^4^ cells/well, respectively. After 24 h, the medium was replaced with the conditioned media (200 µL/well), and cells were treated for 24 h; RPMI+ conditioned without samples was used as negative control. Then, 20 µL/well of the MTT reagent (0.5 mg/mL in PBS) was added, and plates were incubated at 37 °C for additional 3 h. The supernatant was gently removed and replaced with DMSO (100 µL/well) to solubilize the MTT tetrazolium dye. The optical density (OD) was measured at 550 nm wavelength using a microplate reader (Spectrafluor Plus, TECAN Austria GmbH, Grödig, Austria). The percentage of cell viability was calculated vs. that of the negative control, assumed as 100%. Tests were repeated at least three times.

### 4.6. Antibacterial Activity against E. coli

Gram-negative *E. coli* bacterial strain was selected as model organisms to investigate the antimicrobial properties of the produced nanofibrous samples by a direct contact test. Before the test, samples were cut into discs (ø 8 mm) and sterilized under UV light for 1 h per side in a laminar flow cabinet integrated with a UV light source. *E. coli* was cultured in Mueller–Hinton broth at 37 °C. Bacterial suspension was diluted by the colony counting method through ABS measurement at 550 nm. Then, 75 μL of bacterial suspension containing 50 colony-forming units (CFU) were inoculated on the surface of samples. After 2 h at room temperature, the broths were collected and plated on plates containing Mueller–Hinton agar. After additional 24 h of incubation at 37 °C, the number of CFU on each plate was counted and normalized with respect to the control sample, i.e., PU-EM without CuNPs, for which a 100% bacterial viability was assumed. All tests were conducted in duplicate.

### 4.7. Antiviral Activity against SARS-CoV-2

The antiviral properties of sterile PU-EM-CuNPs and PU-EM samples (ø 14 mm) were evaluated as previously reported in [39]. In particular, African Green Monkey Kidney Cells (Vero-E6) and clinical isolate of SARS-CoV-2, kindly gifted from the San Raffaele Hospital (Milan, Italy), were used to perform all experiments. The virus expanded and titrated on VERO E6 cells had a titer of 4.6 × 10^5^ TCID50/mL determined by the Reed and Muench formula and used at 0.1 MOI (multiplicity of infection). The virus inactivation percentage was evaluated at 5, 10, 30, and 60 min of contact. The results obtained were expressed as % inhibition of viral growth, normalizing the values on an infected and untreated cell control. All tests were conducted in triplicate.

### 4.8. Statistical Analysis

Statistically significant differences were determined with GraphPad PrismSoftware 9.5.1, (Boston, MA, USA) through one-way analysis of variance ANOVA. *p* value < 0.05 was considered statistically significant.

## Data Availability

Not applicable.

## Sample Availability

Samples are available upon request.
